# A conceptual study on the relationship between daily stressors, stressful life events, and mental health in refugees using network analysis

**DOI:** 10.3389/fpsyg.2023.1134667

**Published:** 2023-08-03

**Authors:** Malte Behrendt, Marianne Vervliet, Marina Rota, Sarah Adeyinka, Océane Uzureau, Andrew Rasmussen, Heide Glaesmer, Ine Lietaert, Ilse Derluyn

**Affiliations:** ^1^Department of Social Work and Social Pedagogy, Centre for the Social Study of Migration and Refugees, Ghent University, Ghent, Belgium; ^2^Department of People and Well-Being, Thomas More University of Applied Sciences, Mechelen, Belgium; ^3^Culture, Migration, and Community, Department of Psychology, Fordham University, New York, NY, United States; ^4^Department of Medical Psychology and Medical Sociology, Medical Faculty, The University of Leipzig Medical Center, Leipzig, Germany; ^5^Institute on Comparative Regional Integration Studies, United Nations University, Bruges, Belgium

**Keywords:** refugees, migration, mental health, daily stressors, stressful life events, trauma, network analysis, ecological model

## Abstract

**Introduction:**

There is growing recognition that daily stressors, such as social and material deficiencies, can be highly detrimental to the mental health of refugees. These stressors are in addition to stressful life events, which have been widely studied in the context of migration and forced displacement. Despite increasing evidence for an ecological model, there is still no consensus regarding the conceptualization of these highly influential factors. In particular, the demarcation of daily stressors from stressful life events and the categorization of daily stressors require further examination in order to develop usable and accurate tools for researchers, design effective interventions for practitioners and assist politicians in designing meaningful policies.

**Methods:**

To address these challenges, we used data from a sample of 392 unaccompanied young refugees from diverse backgrounds and employed network analysis to examine the relationships between daily stressors, stressful life events, and symptoms of depression, anxiety, and post-traumatic stress.

**Results:**

Our findings highlight the significant relationship between daily stressors and mental health, particularly depression. Meaningful clusters of daily stressors include material stressors, social stressors, and social exclusion stressors.

**Conclusion:**

Our results demonstrate the importance of considering daily stressors in the mental health of refugees and suggest that using a network approach offers a viable way to study these complex interrelationships. These findings have implications for researchers, practitioners, and policymakers in understanding and addressing the mental health needs of refugees.

## 1. Introduction

Refugees^1^ demonstrate remarkable levels of resilience, considering the manifold stressors and challenges they face (Behrendt et al., 2021, 2023). However, research also testifies to the poor mental health status of this group, who consistently show high rates of depression, anxiety and post-traumatic stress symptoms (Morina et al., 2018; Blackmore et al., 2020). These symptoms are often associated with stressful life events (SLEs): potentially traumatic experiences that occur in the context of involuntary migration (Höhne et al., 2020; Behrendt et al., 2022; Pfeiffer et al., 2022; Derluyn et al., 2023). SLEs are typically defined as acute and potentially traumatic events in the past, often in relation to the DSM-5 criterion A for post-traumatic stress disorder (PTSD), namely “exposure to actual or threatened death, serious injury, or sexual violence” (American Psychiatric Association, 2013).

Next, to these experiences, a growing number of studies indicate that daily stressors (DSs) have a major impact on the mental health of refugees (Miller and Rasmussen, 2017; Hajak et al., 2021). In contrast to SLEs, DSs are usually defined as less severe, but on-going and pervasive stressors (Miller and Rasmussen, 2017). In line with a shift from trauma-focused approaches to more ecological approaches, these contextual factors have increasingly received the attention of researchers and practitioners alike (Miller and Rasmussen, 2017; Villanueva O’Driscoll et al., 2017; IOM, 2019). Despite this growing recognition however, there is no consensus with regard to the conceptualization of daily stressors. In the current literature, daily stressors are also called daily hassles or post-migration stressors, even though they may also occur before and during migration. This variety in terms already shows the lack of a coherent concept. Two major issues that researchers are currently struggling with are the demarcation from the more established concept of SLEs and the categorization of the various DSs.

The relationship between DSs, SLEs and mental health outcomes has been discussed at length (Neuner, 2010; Miller and Rasmussen, 2014; Riley et al., 2017). Different pathways have been proposed and according to the model developed by Miller and Rasmussen (2010, 2014, 2017), DSs can affect mental health either directly, as they are emotionally taxing, or indirectly, by depleting coping resources and mediating the effects of SLEs on mental health. Recent evidence supports the idea that there is indeed a qualitative difference between SLEs and DSs (Behrendt et al., 2023). However, it is quite difficult to distinguish these two kinds of stressors and to identify pathways toward the resulting mental health symptoms, also because there is often an interaction effect between them (Miller and Rasmussen, 2014; Mootoo et al., 2019). This is problematic because the oftentimes scarce resources in terms of humanitarian aid require a prioritization of different approaches to treatment such as trauma-focused approaches or interventions primarily focused on social stressors (Silove et al., 2017). Further, refugees may adopt specific coping strategies for SLEs and DSs, and practitioners working with them need to design appropriate interventions depending on the stressors they face (Kocijan-Hercigonja et al., 2009; Behrendt et al., 2023).

Another critical issue is the categorization of different daily stressors. They are usually defined quite broadly and are thought to include a wide range of variables ranging from material stressors, liminal contexts due to temporary legal status, acculturation stress and discrimination to the loss of social networks (e.g., Vervliet et al., 2014). However, most studies examining the impact of daily stressors on the mental health of refugees do not distinguish these diverging kinds of stressors at all. Other studies focus exclusively on single variables, such as the disruption of social networks (Sierau et al., 2019), the asylum process (Jakobsen et al., 2017), or placement in large-scale centers (O’Higgins et al., 2018), thus disregarding other stressors. As Rasmussen and Jayawickreme (2020) point out, there is a need for the revision of tools and methodology in the refugee mental health field. Yet, we lack a clear-cut and meaningful conceptualization of DSs, which is critical for the development of tools that allow an accurate assessment.

Attempts to conceptualize and demarcate stressors following exposure to political violence are often made at the expense of methodological rigor (Netland, 2005). In order to tackle the challenges mentioned above, researchers who strive to examine DSs in a more comprehensive way often resort to the use of sum scores, scales that are not yet validated (e.g., Vervliet et al., 2014; Jensen et al., 2019; Müller et al., 2019) or the use of measures that are not adapted to specific populations (e.g., Seglem et al., 2014; Ponnamperuma and Nicolson, 2018). As Mootoo et al. (2019) illustrate, the use of composite scores has limited value for practitioners who need to understand specific aspects of stress factors in order to design interventions accordingly.

Furthermore, researchers who attempt to group daily stressors often do so by applying factor analysis (e.g., Keles et al., 2017; Müller et al., 2019), even though the validity of this method has been contested (Netland, 2005; De Schryver et al., 2015; Rasmussen et al., 2018). This is because it presupposes that indicators represent latent constructs, in line with the dominant “reflective model” in psychology, e.g., observable symptoms reflect an underlying and essential attribute such as depression (Schmittmann et al., 2013). However, DSs and SLEs are concrete, real-life events or conditions, not latent: Items on event checklists correlate with one another, rather than with an underlying construct. Therefore, Netland (2005) argues that this conventional method of categorizing them is inappropriate.

To circumvent this pitfall, network analysis has been proposed as a possible alternative to analyze the complex relationships between stressors and mental health outcomes (De Schryver et al., 2015; Mootoo et al., 2019). Network analysis is a promising and future-oriented method that has recently gained widespread appreciation in the mental health field (Schmittmann et al., 2013; Hevey, 2018; Birkeland et al., 2020). This method does justice to the complex interrelationships of variables and different factors inherent to the migration context (De Schryver et al., 2015; Morvan et al., 2020). It recognizes that different stressors and mental health symptoms are causally interrelated, and allows to investigate how constructs relate to one another and how specific indicators influence constructs (De Schryver et al., 2015; Mootoo et al., 2019).

The aim of the current study is to nuance and flesh out existing concepts of DSs in order to address the lack of conceptual clarity in the field and contribute to the development of more precise and valid tools. Using a network approach, we examine the relationships between DSs, SLEs and the mental health outcomes depression, anxiety and PTSD to answer the research questions:

1. How are DSs different from SLEs?
2. How can DSs be grouped in a meaningful way?

These questions are examined in a sample of unaccompanied young refugees (UYRs), defined as young people who feel forced to leave their home country unaccompanied by their parents or other relatives and who are not being cared for by an adult who, by law or custom, is responsible for doing so (UNHCR, Unicef, and IOM et al., 2019). This group is representative of the population of refugees with documented histories of DSs and SLEs that lead to severe mental health symptoms including anxiety, depression and post-traumatic stress (Behrendt et al., 2022; Pfeiffer et al., 2022). In addition to the stress associated with forced migration, they can be seen as particularly vulnerable because they lack the parental, social (and material) support that is crucial for this age group (Behrendt et al., 2021; Pfeiffer et al., 2022). As in the general population, the extent to which UYRs who experience SLEs develop PTSD varies considerably (Van der Kolk, 2000) and El-Awad et al. (2021) illustrate the differences between unaccompanied and accompanied young refugees and other migrant groups with regard to the complex pathways between stressors and mental health outcomes. However, UYRs show an increased prevalence of PTSD in comparison (Ehntholt et al., 2018; Bamford et al., 2021) and their heightened susceptibility to both SLEs and DSs makes them a suitable population for this study. Whereas our research project used a mixed-methods design and the narrative accounts of the participants would have added value to the quantitative measures, the inclusion of these qualitative data was beyond the scope of this conceptual study, which focuses on self-reported quantitative data. For in-depth analyses of our qualitative data, the reader is referred to our other publications (Derluyn et al., 2022).

## 2. Methods

### 2.1. Participants and setting

Data were drawn from two similar studies. First, we used the data from the ChildMove project, a research project investigating the impact of flight experiences on the psychological health of UYRs (Derluyn et al., 2022). Participants were recruited in Libya (*n* = 100), Greece (*n* = 45), Italy (*n* = 65), and Belgium (*n* = 79). Second, we used data from a previous comparable study with URMs in Belgium (*n* = 103) (Vervliet et al., 2014), yielding a total of *N* = 392 participants. Both studies were longitudinal in nature, yet here we only used the data from the baseline measurement because of considerable attrition over time. Participants were 16 years old on average (*M* = 16.19, SD = 1.87), most were male (84%), and the top five countries of origin were Eritrea (*n* = 72), Afghanistan (*n* = 34), Nigeria (*n* = 31), Somalia (*n* = 29), and Pakistan (*n* = 21).

We recruited the participants in a variety of settings, including migrant detention facilities, official reception facilities, NGO shelters and informal settings. The researchers strived to build rapport with the participants and gave a detailed explanation of the research before asking them to take part. We selected participants to represent the population of UYRs with regard to their nationality, age and gender in each setting. Before each interview, we reminded the youth that their participation in the research was voluntary, that we would treat the data confidentially and would anonymize them, that there would be no consequences for them (e.g., with regard to their legal procedures) and that they could stop their participation at any time. Cultural mediators assisted in the recruitment and interview process according to the participants’ preference. In preparation for the field work, we had established a referral network in each study setting to guide participants to social, legal and mental health care providers if need be. All participants and their guardians (if they had one) gave written consent for their participation. The Committee of Ethics in Research at the University of West Attica, the Hellenic Data Protection Authority, the Italian National Research Council’s Committee on Research Ethics and Bioethics (0059862) and the ethics committee of the Faculty of Psychology and Educational Sciences at Ghent University (#2017-23-Ine Lietaert) gave their approval for the respective studies.

### 2.2. Measures

We operationalized the concepts under study with the following questionnaires. All questionnaires were translated into the languages present in the population (including Albanian, Amharic, Arabic, Dari/Farsi, English, French, Pashto, Servo-Croatian, Somali, Tigrinya and Urdu). If it turned out to be necessary, the translations were revised by a second translator.

Daily Stressors Scale for Young Refugees (DSSYR) (Vervliet, 2013). This 15-item questionnaire assessed daily stressors in the 4 weeks prior to the interview. We omitted items 2 (“Difficulties in relationships with adults”), 3 (“Difficulties in relationships with youngsters”) and 5 (“Other difficulties in the family”) because we queried these items extensively in the qualitative interviews. In the samples from the ChildMove project, item 11 was changed to “feeling bored” as this is supposedly easier to understand than “Having no satisfaction with how free-time is filled in.” Likewise, item 5 [“Other difficulties in the family (such as an illness)”] was changed to “Worrying about my family at home” for participants of the ChildMove project (see Table 1). Participants answered each item on a 4-point Likert scale (never, sometimes, often, always) and also had the option to indicate “I do not know/I do not want to answer.” Table 1 shows the items of the Daily Stressors Scale for Young Refugees, as used in both studies.

**Table 1:** Items of the Daily Stressors Scale for Young Refugees.

#	Item	*M*	SD
1	Not enough food/clothing	2.16	1.04
2	Not enough money	2.56	1.25
3	Not enough housing	1.96	1.22
4	Not enough medical care	1.78	1.13
5	Feelings of unsafety	1.85	1.06
6	Difficulties in making new friends	1.80	1.09
7	Worrying about my family at home^a^ Other difficulties in the family (such as an illness)^b^	2.97	1.20
8	Difficulties in obtaining legal documents	2.27	1.21
9	Feeling bored^a^ Having no satisfaction with how free-time is filled in^b^	2.15	1.04
10	Feeling uncertain about the future	2.32	1.06
11	Hear people say bad things about myself	1.38	0.69
12	Feeling of being treated unfairly compared to others	1.44	0.80
13	Feeling that others have prejudices about myself or people of my country/culture	1.68	1.03

^a^Wording in ChildMove study, ^b^Wording in Vervliet et al.’s (2014) study, Range = 1–4.

Stressful Life Events (SLE) (Bean et al., 2004). This 12-item questionnaire measures if and how many traumatic events the participants witnessed throughout their lives. For the participants of the ChildMove project, we changed item 1 from “Have there been drastic changes in your family during the last year?” to “Have there been drastic changes in your family?” in order to include events over the course of their entire lives rather than just during the last year. Items 3 (“Has someone died in your life who you really cared about?”), 4 (“Have you had a life-threatening medical problem?”), 5 [“Have you been involved in a serious accident (for example involving a car)?”] and 6 [“Have you ever been involved in a disaster (for example: flood, hurricane, fire, tornado, avalanche, earthquake, hostage situation, chemical disaster,…)?”] have been omitted because they were covered sufficiently by the other items resulting in a limited amount of questions as the interviews tended to be too long and exhausting for the participants. Using only the items common to both studies, eight items remained. Table 2 shows the items of the Stressful Life Events questionnaire, as used in both studies.

**Table 2:** Items of the Stressful Life Events.

#	Item	Endorsed (%)
1	Have there been drastic changes in your family?^a^ Have there been drastic changes in your family during last year?^b^	65.2
2	Have you ever been separated from your family against your will? (By a stranger, police officer, soldier, fleeing your country of origin)	40.3
3	Have you ever experienced a war or armed military conflict going on around you in your country of origin?	61.8
4	Has someone ever hit, kicked, shot or some other way tried to physically hurt you?	76.0
5	Did you ever see it happen to someone else in real life (not just on television or in a film)?	87.6
6	Has someone ever tried to touch your private sexual parts against your will or forced you to have sex?	23.6
7	Did you experience any other very stressful life events where you thought that you were in great danger?	88.0
8	Did you experience any other very stressful life events where you thought that someone else was in great danger?	79.4

^a^Wording in ChildMove study, ^b^Wording in Vervliet et al.’s (2014) study.

Hopkins Symptom Checklist-37A (HSCL) (Bean et al., 2007). This 37-item questionnaire builds on and adds 12 externalizing items to the HSCL-25 in order to adapt it to adolescents from diverse backgrounds. Next to these externalizing symptoms, it assesses anxiety and depression symptoms in the 4 weeks prior to the interview. Participants answered each item on a 4-point Likert scale (never, sometimes, often, always) and also had the option to indicate “I do not know/I do not want to answer.” The anxiety subscale included items 1, 2, 5, 8, 11, 14, 17, 20, 23, and 26. The depression subscale included items 6, 9, 13, 15, 18, 21, 22, 24, 27–30, 32, and 33.

Reactions of Adolescents to Traumatic Stress (RATS) (Bean et al., 2006). This 22-item questionnaire measures PTSD symptoms according to the DSM-IV. Same as with the previous questionnaires, we used an abbreviated version of the RATS in order to reduce the potential burden of trauma assessment and omitted the items that had been assessed in Vervliet et al.’s (2014) study but not in the ChildMove study. Participants answered each item on a 4-point Likert scale (never, sometimes, often, always). The measure includes the three subscales intrusion (items 1, 2, 3, 4, and 5), numbing/avoidance (items 6, 7, and 8) and hyperarousal (items 9 and 10).

### 2.3. Data analysis

First, the ordinal HSCL and RATS scales were transformed to the continuous scores of their subscales. The nominal answer categories of the SLE (where the event had taken place) have been collapsed into whether or not participants had experienced the event in their lifetime. The answer categories of the DSSYR remained ordinal. After listwise deletion of cases with missing values, *N* = 233 (59.4%) cases remained. In order to find out about the roles of SLEs and DSs in relation to the construct of post-traumatic stress disorder and mental health in general, we calculated two network models: One with the items of the DSSYR, SLE and the HSCL subscales anxiety and depression (23 variables), and one with the items of DSSYR, SLE and the RATS subscales avoidance, intrusion and hyperarousal (24 variables). For both models, we used the estimateNetwork function in the bootnet package with graphical least absolute shrinkage and selection operator (GLASSO) regularization, resulting in a collection of more parsimonious and more easily interpretable networks. We then selected the best network using the Extended Bayesian Information Criterion (EBIC) with the tuning-parameter set to 0.5 (Epskamp et al., 2018). Given the use of different data sets and the exclusive focus on the first measurement moment of the longitudinal studies, we controlled for the potentially confounding effects of data set and measurement moment. To this end, we calculated and compared models for each subsample and the first and last time points, but found no systematic differences between them.

Next, we investigated the quality of the connections between the nodes. We calculated the following centrality values for both models using the R package qgraph (Epskamp et al., 2012): strength (the degree to which a node correlates with all other nodes in the network, indicating how strongly they are connected), expected influence (the sum of correlation coefficients that are either positive or negative, indicating the degree to which a node affects other nodes), betweenness (the degree to which a node lies on the shortest path between two other nodes, indicating the control a node has), and closeness (the inverse sum of the shortest distances to all other nodes, indicating indirect connection to all other nodes). Following the recommendations of Epskamp et al. (2018), we estimated the accuracy of edge weights by means of a nonparametric bootstrap procedure with 2,500 samples, and tested the stability of centrality indices using a case-dropping subset bootstrap framework.

Finally, we used the R package blockmodels (Leger et al., 2021) in R 4.0.2 (R Core Team, 2020) to perform stochastic blockmodeling (Holland et al., 1983) to estimate and visualize the final models. Blockmodeling clusters nodes that have similar relationships with all other nodes in the network in the same block, based on their so-called structural equivalence. Each block, or cluster of nodes, thus represents a different role in the network, which provides important information about their significance in relation to other nodes. In our case, this means that stressors, events or mental health outcomes associated with the same cluster play a similar role with respect to other stressors, events or mental health outcomes.

## 3. Results

### 3.1. Descriptive statistics

Table 1 shows the items of the DSSYR and their mean ratings. Participants were particularly concerned about their family, not having enough money and feeling uncertain about the future. Table 2 shows the items of the SLE, along with the percentage of participants who endorsed each item. The most commonly reported stressful life events were experiencing a very stressful life event where they thought that they were in great danger (88.0%), witnessing physical violence (87.6%) and witnessing a very stressful life event where they thought that someone else was in great danger (79.4%). Table 3 shows the mean ratings of internalizing symptoms (HSCL) and PTSD symptoms (RATS).

**Table 3:** Endorsement of HSCL and RATS symptoms.

	Mean	SD	Range
HSCL			
Anxiety	1.90	0.55	1.00–3.80
Depression	2.06	0.56	1.00–3.43
RATS			
Avoidance	2.92	0.92	1.00–4.00
Intrusion	2.38	0.73	1.00–4.00
Hyperarousal	2.52	0.86	1.00–4.00

### 3.2. Network accuracy and stability

Supplementary Figures S1, S2 show the results of the tests for network edge accuracy. Confidence intervals were generally broad and overlapping, indicating that the rank order of edge values should be interpreted with caution. Supplementary Figures S3, S4 show the results of the tests for network stability with a centrality stability coefficient of 0.7 (indicating the estimated maximum number of cases that can be removed from the data to maintain a correlation of at least 0.7 with 95% probability). For model 1, the correlation stability coefficient was 0.129 for betweenness, 0 for closeness and 0.438 for strength. For model 2, it was 0 for betweenness, 0 for closeness and 0.361 for strength. Therefore, the centrality rank order of betweenness and closeness is not stable enough to be interpreted, the centrality difference of strength is the only one that should be interpreted in the two models.

### 3.3. Network visualization

**Figure 1:**
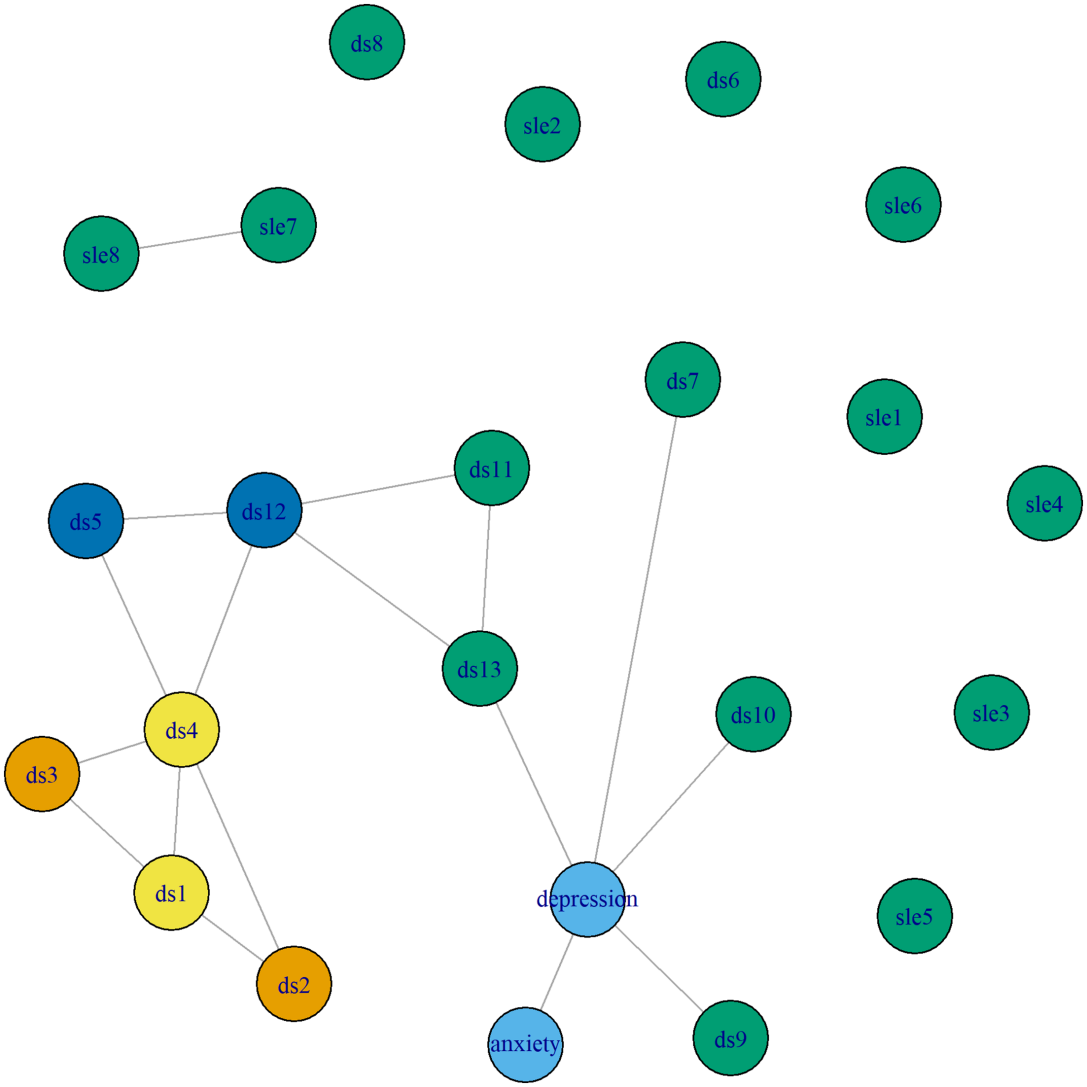
Network model 1 with the HSCL subscales anxiety and depression.

**Figure 2:**
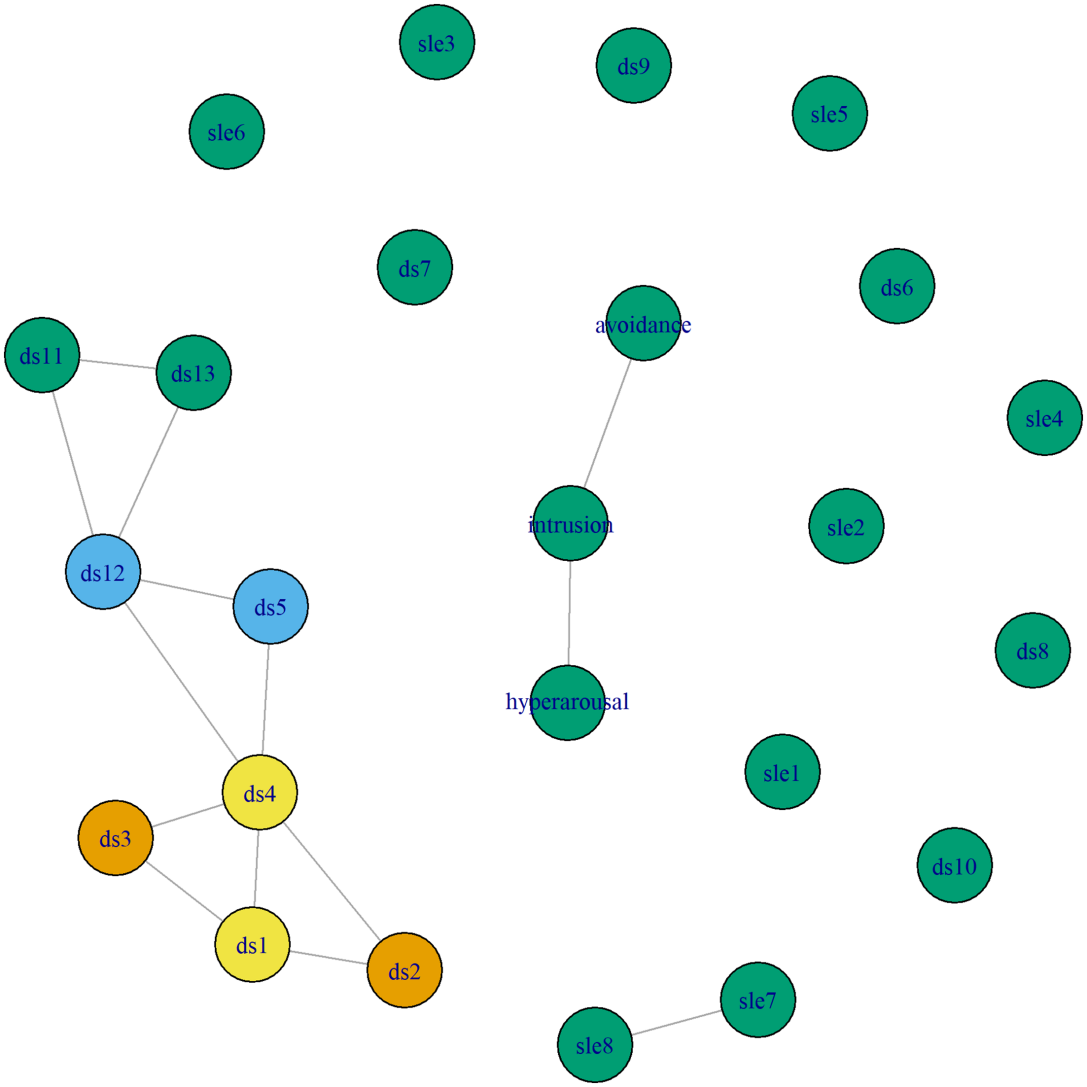
Network model 2 with the RATS subscales avoidance, intrusion and hyperarousal.

The visualized network models are presented in Figure 1 (model 1 with the HSCL subscales anxiety and depression) and Figure 2 (model 2 with the RATS subscales avoidance, intrusion and hyperarousal). Different colors indicate different clusters of nodes that share a similar role in the network. Edge weight ranged from 0.008 (ds7 – depression) to 0.346 (anxiety – depression) in model 1 and from 0.014 (ds11 – ds12) to 0.231 (ds1 – ds4) in model 2. Model 1 had a density of 0.927 (246/253 edges) and a mean weight of 0.164 and model 2 had a density of 0.902 (249/276 edges) and a mean weight of 0.118.

There are 5 clusters in model 1 and 4 clusters in model 2. Both figures show that these include two clearly identifiable clusters of material stressors (DSs items 1–4). There also seems to be a cluster of items that represent discrimination and stigmatization (DSs items 5 and 12). Interestingly, a “feeling of being treated unfairly compared to others” (DSs item 12) has a similar role as “feelings of unsafety” (DSs item 5) in relation to other stressors, events or mental health outcomes. Comparing both models, results indicate that while symptoms of anxiety and depression play a distinct role in relation to DSs and SLEs, the PTSD symptoms avoidance, intrusion and hyperarousal do not play a distinct role in this context, as they were assigned the same cluster as all SLEs and some DSs. Finally, the network structures and clusters show that the remaining daily stressors have the same role in the network as stressful life events and post-traumatic stress symptoms, and that they are not necessarily associated with mental health outcomes. However, DSs items 7, 9, 10, and 13 do correlate with depression.

### 3.4. Centrality indices

**Figure 3:**
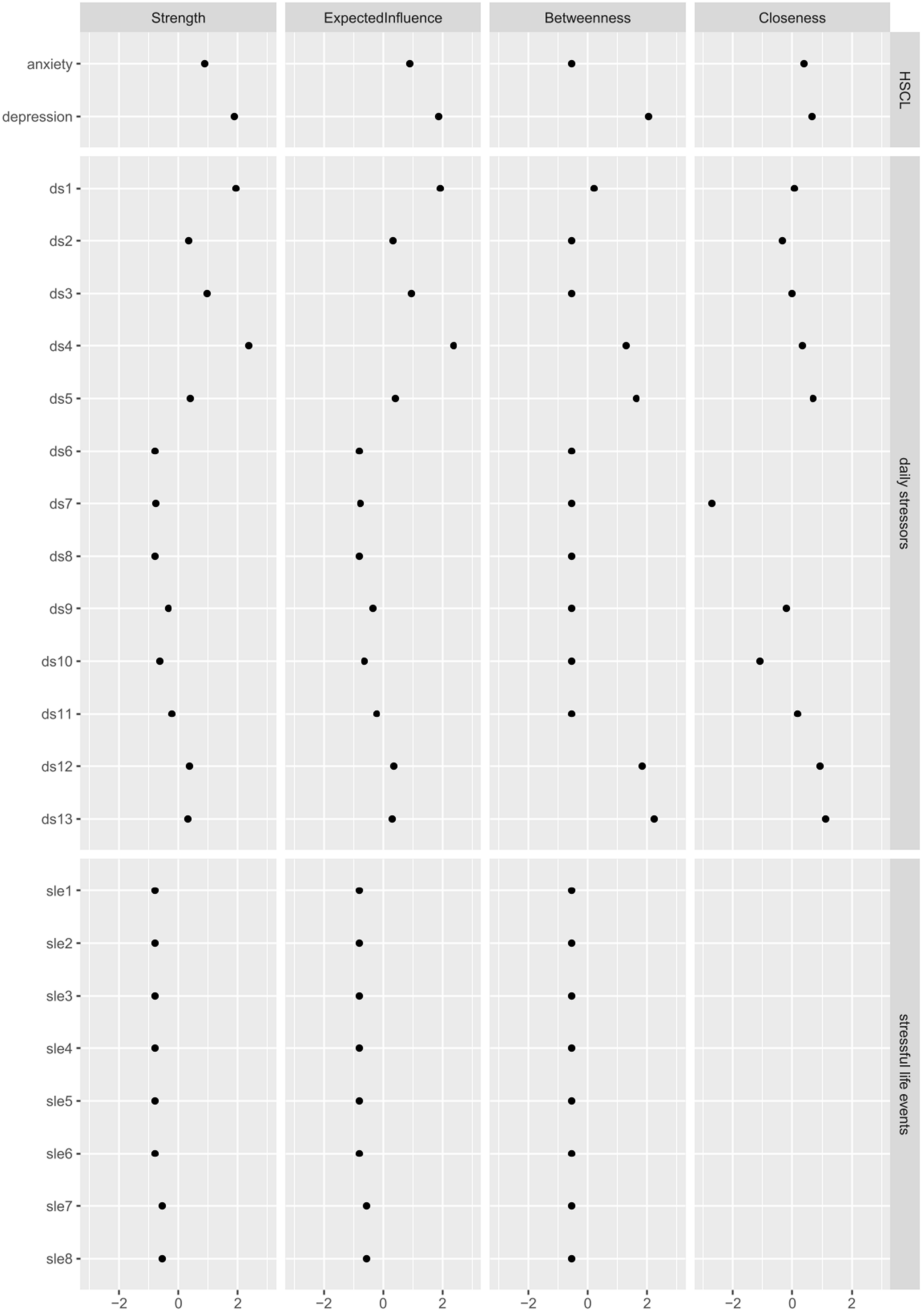
Standardized centrality values for model 1.

**Figure 4:**
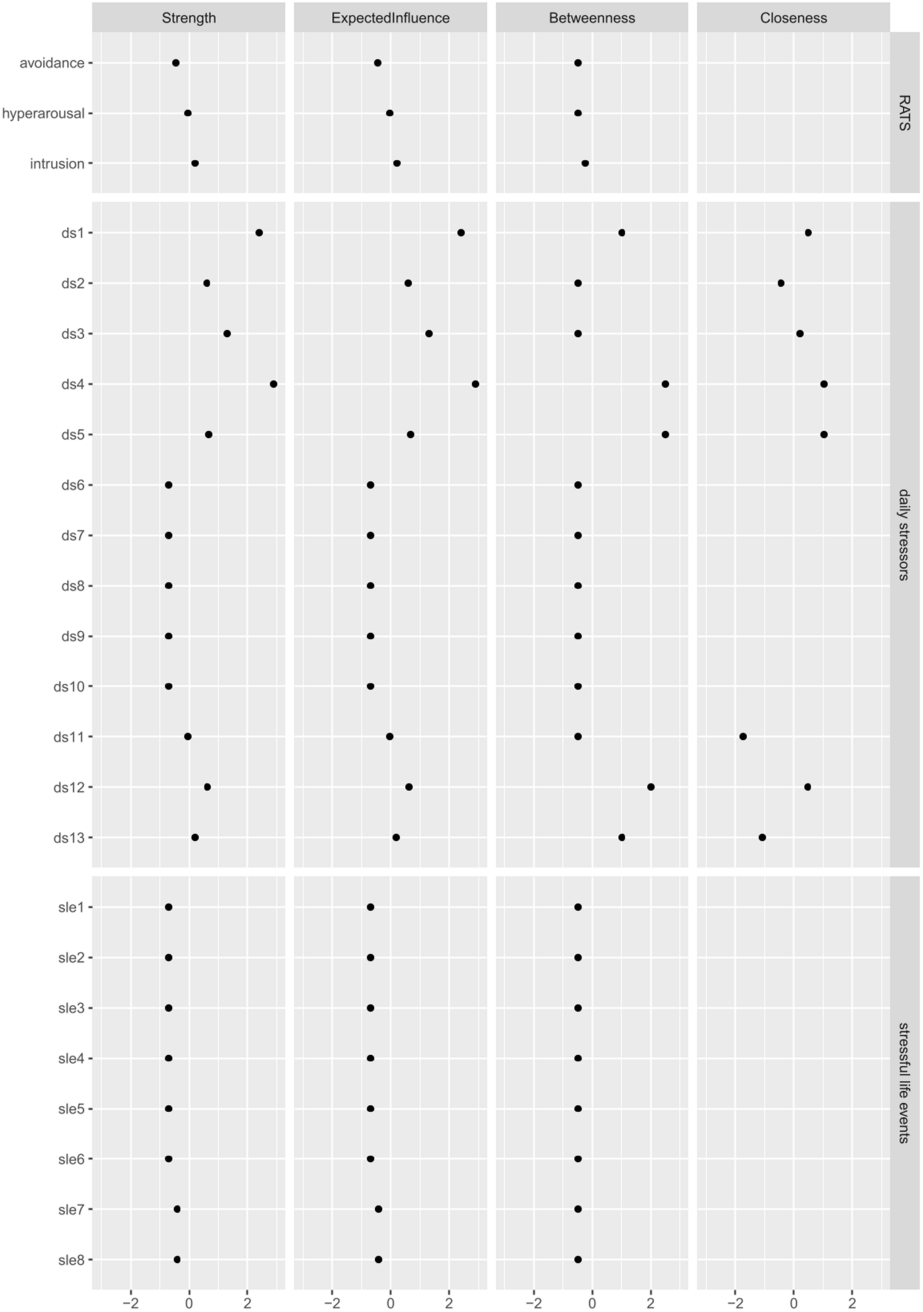
Standardized centrality values for model 2.

Tables 4, 5 show mean centrality values by node type and Figures 3, 4 show the standardized centrality values per item and by node type for model 1 and 2, respectively. Same as the visualization, these strength centrality values indicate that depression, anxiety and daily stressors (particularly material and discrimination stressors) are most strongly connected and play a more central role than other stressors.

**Table 4:** Mean centrality values by node type for model 1.

	Betweenness	Closeness	Strength	Expected influence
Depression	0.927	0.911	0.846	0.846
Anxiety	0.000	0.856	0.533	0.533
Daily stressors	0.274	0.758	0.328	0.328
Stressful life events	0.000		0.020	0.020

**Table 5:** Mean centrality values by node type for model 2.

	Betweenness	Closeness	Strength	Expected influence
Avoidance	0.000		0.067	0.067
Hyperarousal	0.000		0.183	0.183
Intrusion	0.083		0.249	0.249
Daily stressors	0.295	0.797	0.304	0.304
Stressful life events	0.000		0.019	0.019

## 4. Discussion

In this study, we set out to explore and add nuance to the concept of DSs for the population of refugees, which is critical to further optimize treatment and care. Below, we will discuss our results with regard to how DSs differ from SLEs and how DSs can be grouped in a meaningful way. At the same time, we will discuss some practical and theoretical implications.

The concept of PTSD has proven to be useful for diagnosis and treatment and has led to a proliferation of evidence-based therapies (Bisson and Olff, 2021). Yet, it is still contested and criticized for various reasons. For instance, a number of comorbidities typically accompany PTSD diagnoses, such as somatic symptom disorder, depression or substance abuse (Nesterko et al., 2020; Brady et al., 2021; Im et al., 2022; Kratzer et al., 2022). Further, there is a variety of trauma-related disorders including adjustment disorder and acute stress disorder, as well as others, such as borderline personality disorder, that have been linked to adverse childhood experiences (sexual abuse in particular) (Oral et al., 2016). With special relevance for refugees, the concept of PTSD neglects the role of daily stressors which may perpetuate or trigger trauma reactions due to their chronic nature (Maercker et al., 2018).

Consistent with this critique and the ecological model of refugee distress (Miller and Rasmussen, 2017), our results show that daily stressors generally have a significant relationship with mental health outcomes (specifically depression), but correlate much less with the RATS subscales avoidance, intrusion and hyperarousal. SLEs, on the other hand, are much less connected to anxiety and depression symptoms. The fact that anxiety and depression are related to DSs but not to SLEs suggests that DSs are highly significant for measures of internalizing symptoms. This confirms previous research (Jayawickreme et al., 2017; Mootoo et al., 2019) and goes to show that refugees may suffer from mental health symptoms even when they fail to reach the “trauma threshold” (Van der Kolk, 2000; De Schryver et al., 2015). Although scales measuring post-traumatic stress are essential for individuals with clinical symptom levels, we can deduce that dominant concepts such as PTSD fail to capture the complete range of relevant factors for the study or assessment of mental distress in this population (Miller and Rasmussen, 2017; Villanueva O’Driscoll et al., 2017). Considering this, the way PTSD is conceptualized now is too narrow, and, as Silove et al. (2017) have suggested, the range of mental health outcomes resulting from forced displacement can be viewed as a continuum rather than distinct and arbitrary categories.

Specifically, the fact that material (DSs items 1–4) and some discrimination stressors (DSs items 5 and 12) play a distinctive role in relation to other stressors and mental health outcomes as well as their correlation with depression suggest that they play a prominent role with important implications for therapy and treatment. Often, DSs are seen as obstacles for trauma treatment, rather than as a direct source of psychological suffering and not as a focus of intervention in their own right. In this regard, the centrality of these factors underlines the relevance of stepped care approaches and suggests that psychiatric treatment may benefit from the integration of community care and social work (Fazel and Betancourt, 2018).

For practitioners, this implies that psychosocial interventions or transdiagnostic approaches (Murray et al., 2014) are warranted in addition to trauma-focused approaches. Further, the divergence between DSs and SLEs in our results suggests that practitioners need to support specific coping strategies for specific stressors (Behrendt et al., 2023). Depending on the different kinds of stressors at play, refugees may benefit more from particular types and sources of support (Kocijan-Hercigonja et al., 2009). For example, the study by Goodkind et al. (2014) provides an illustration of an intervention tackling social isolation. Finally, tackling daily stressors directly, including the precarious living conditions in both transit and settlement situations, is key to improve the mental health of refugees.

The second aim of this study was to investigate how different types of daily stressors can be grouped meaningfully. In line with the scale measuring post-migration stress in adult refugees developed recently by Malm et al. (2020), we found a cluster of material stressors, as well as a cluster of discrimination stressors. Our finding that discrimination stressors formed a distinctive category also aligns with the results of Keles et al. (2017), who describe a difference between daily stressors in the general population (e.g., interpersonal conflicts) and stressors that are specific to the migration and acculturation context (e.g., discrimination). They suggest that the latter have a direct effect on depression in UYRs, whereas the relationship between general daily stress and depression is more reciprocal. Further, the fact that in our network model, experiencing discrimination has the same role as feeling unsafe further illustrates the drastic consequences of social exclusion on mental health (Saasa et al., 2021).

Our items “worries about the family,” as well as “feeling bored” and “uncertainty about the future” all correlated strongly with depression. They correspond with the dimensions “family and home country concerns” and “social strain” in the scale developed by Malm et al. (2020) and with the category “stressors related to the asylum process and visa status” in the scale used by Carlsson and Sonne (2018). Their heightened significance makes sense considering the impact of family support and legal status documented by previous research (Jakobsen et al., 2017; Derluyn and Ang, 2020). Arguably, these items have a social dimension in common and might therefore be grouped as social stressors, which have also been found to be central factors in the study by Jayawickreme et al. (2017). Whereas they were not clustered as a distinctive group in our network model, the clusters social stressors and material stressors did become apparent in studies using factor analysis and our findings seem to validate these previous attempts of categorizing DSs (Malm et al., 2020; Behrendt et al., 2022; Pfeiffer et al., 2022). Therefore, scales measuring DSs need to comprise subscales for material stressors and social stressors, specifically social exclusion stressors.

### 4.1. Limitations

Some limitations deserve our attention. The inclusion of diverging (sub)samples provided a sufficiently large sample to conduct a network analysis (Epskamp et al., 2018; Schönfelder et al., 2021). However, the diverse settings in which our participants were recruited complicate a straightforward analysis, as participants may have experienced different kinds and intensities of DSs in different study countries. Further, we may not have been able to detect DSs that occur after arrival in a host country (e.g., DSs item 8 “difficulties in obtaining legal documents”) as many participants were still on the move. Similarly, there was limited assessment of DSs pre- and peri-migration in some subsamples. These missing values may have skewed our results as they may have concerned a particularly vulnerable subgroup of our sample. In contrast, it is possible that some stressful life events showed little variance because of a ceiling effect. Other confounding variables that are closely linked to DSs but were not regarded in this study include legal status and social support, e.g., whether or not participants had contact with their parents. Another point of attention is that some items that we had added in the ChildMove study had to be omitted here in order to allow for the inclusion of the additional dataset. Some of these items turned out to be quite highly reported (e.g., “difficulties to communicate with others due to the foreign language”). The language barrier is therefore a potentially important DS. Related to this, the participants’ cultural background and cultural fit with the host society may have implications for their perception of DSs. Future studies may remedy these shortcomings by collecting more data to compare different study contexts and control for these variables or selecting more homogeneous samples. Finally, the field may benefit from an analysis of the content overlap of the scales we used, similar to that examined by Fried et al. (2022) in the context of depression.

## 5. Conclusion

This study is one of the first to employ network analysis to study the concept of DSs and has allowed us to confirm and expand previous studies (De Schryver et al., 2015; Rasmussen et al., 2018; Mootoo et al., 2019). Our results legitimize previous findings and may be interpreted as evidence that while methodologically controversial approaches such as factor analysis can be useful to explore the nature and possible categorization of DSs, using a network approach offers a viable way forward. Although more research is needed to further flesh out the various subgroups, our study suggests that practitioners, policy-makers and researchers need to direct their attention to both material and social determinants of refugees’ mental health. This is particularly relevant against the background of negative political attitudes toward refugees in host countries and a deteriorating human rights situation in Europe (Council of Europe, 2019). Next to improving the precarious living conditions for this population, practitioners and policy-makers alike urgently need to tackle racism and promote social inclusion and support.

## Data availability statement

The raw data supporting the conclusions of this article will be made available by the authors, without undue reservation.

## Ethics statement

The studies involving human participants were reviewed and approved by the Committee of Ethics in Research at the University of West Attica, the Hellenic Data Protection Authority, the Italian National Research Council’s Committee on Research Ethics and Bioethics and the Ethics committee of the Faculty of Psychology and Educational Sciences at Ghent University. Written informed consent to participate in this study was provided by the participants’ legal guardian/next of kin.

## Author contributions

MB, AR, HG, and ID contributed to conception and design of the study. MB, MV, MR, SA, and OU collected the data. MB organized the database, performed the statistical analysis, and wrote the first draft of the manuscript. All authors contributed to manuscript revision, read, and approved the submitted version.

## Funding

The ChildMove project was funded by the European Research Council (HORIZON project number: 714222).

## Conflict of interest

The authors declare that the research was conducted in the absence of any commercial or financial relationships that could be construed as a potential conflict of interest.

## Publisher’s note

All claims expressed in this article are solely those of the authors and do not necessarily represent those of their affiliated organizations, or those of the publisher, the editors and the reviewers. Any product that may be evaluated in this article, or claim that may be made by its manufacturer, is not guaranteed or endorsed by the publisher.

## Footnotes

^1^As the line between the categories of “migrants” and “refugees” is often blurred (Derluyn and Vervliet, 2012; Lorenzen, 2018), we use a social definition of refugee to include all individuals who feel forced to flee their home country, as well as those who have applied for international protection.

## Supplementary material

Supplementary FIGURE S1 Results of network edge accuracy analyses for model 1.

Supplementary FIGURE S2 Results of network edge accuracy analyses for model 2.

Supplementary FIGURE S3 Results of network stability analyses for model 1.

Supplementary FIGURE S4 Results of network stability analyses for model 2.
