# Supplementary figures and images for: A conceptual study on the relationship between daily stressors, stressful life events, and mental health in refugees using network analysis
Source: Front Psychol. 2023 Aug 3;14:1134667. doi: 10.3389/fpsyg.2023.1134667 (PMC10438848; doi:10.3389/fpsyg.2023.1134667)

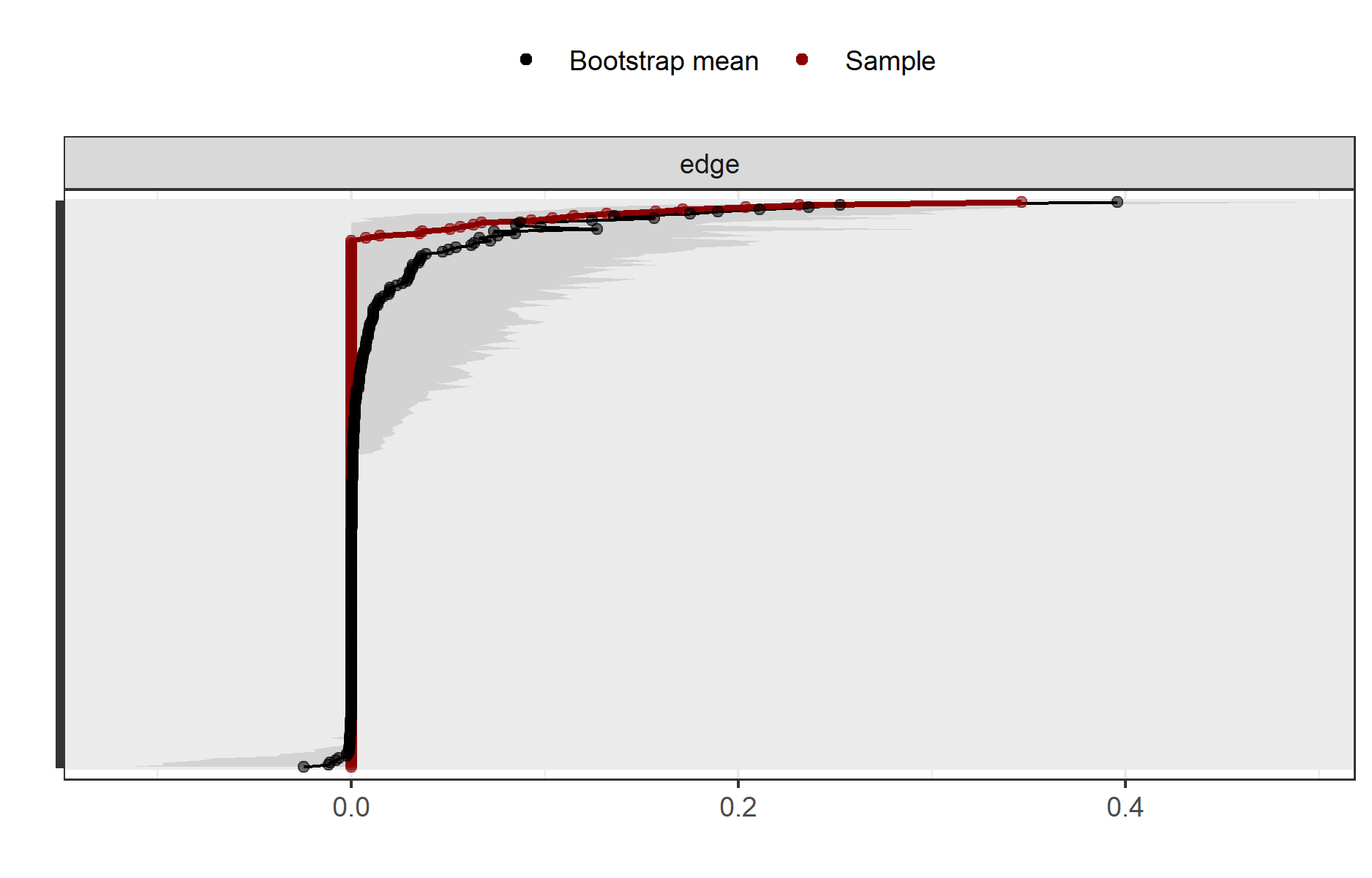

Supplement: Supplementary FIGURE S1 — Results of network edge accuracy analyses for model 1. [file Image_1.tiff]

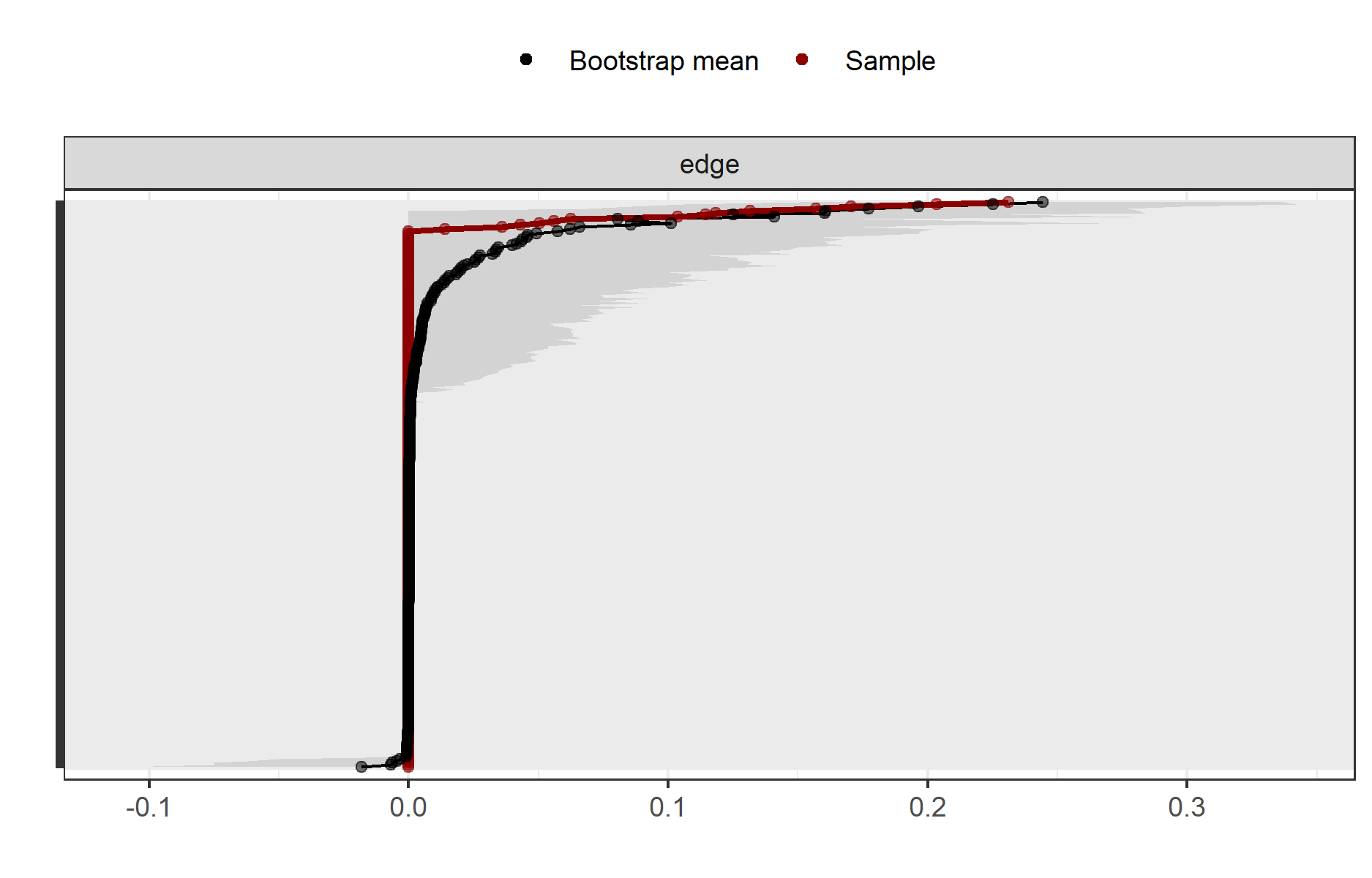

Supplement: Supplementary FIGURE S2 — Results of network edge accuracy analyses for model 2. [file Image_2.tiff]

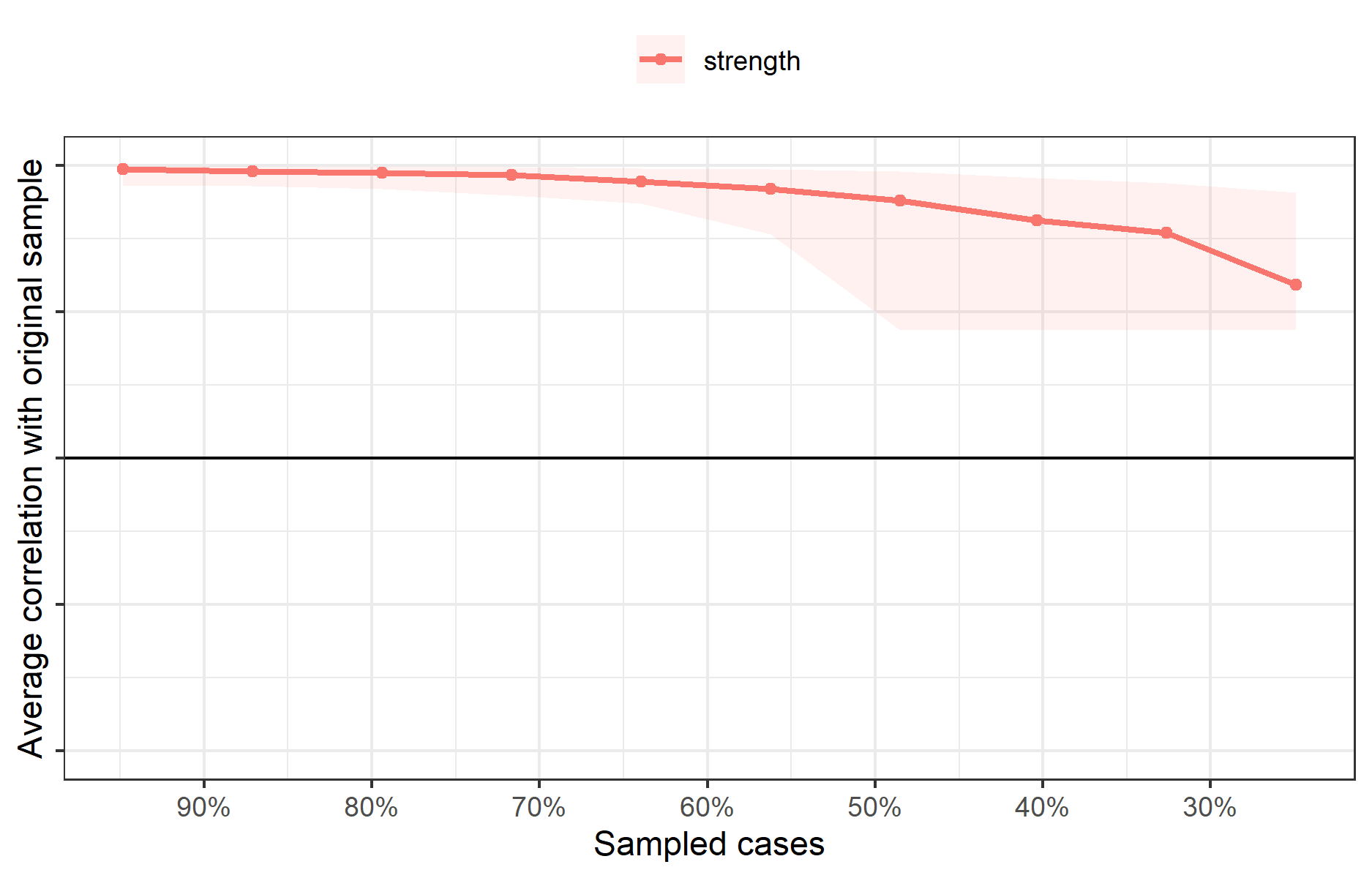

Supplement: Supplementary FIGURE S3 — Results of network stability analyses for model 1. [file Image_3.tiff]

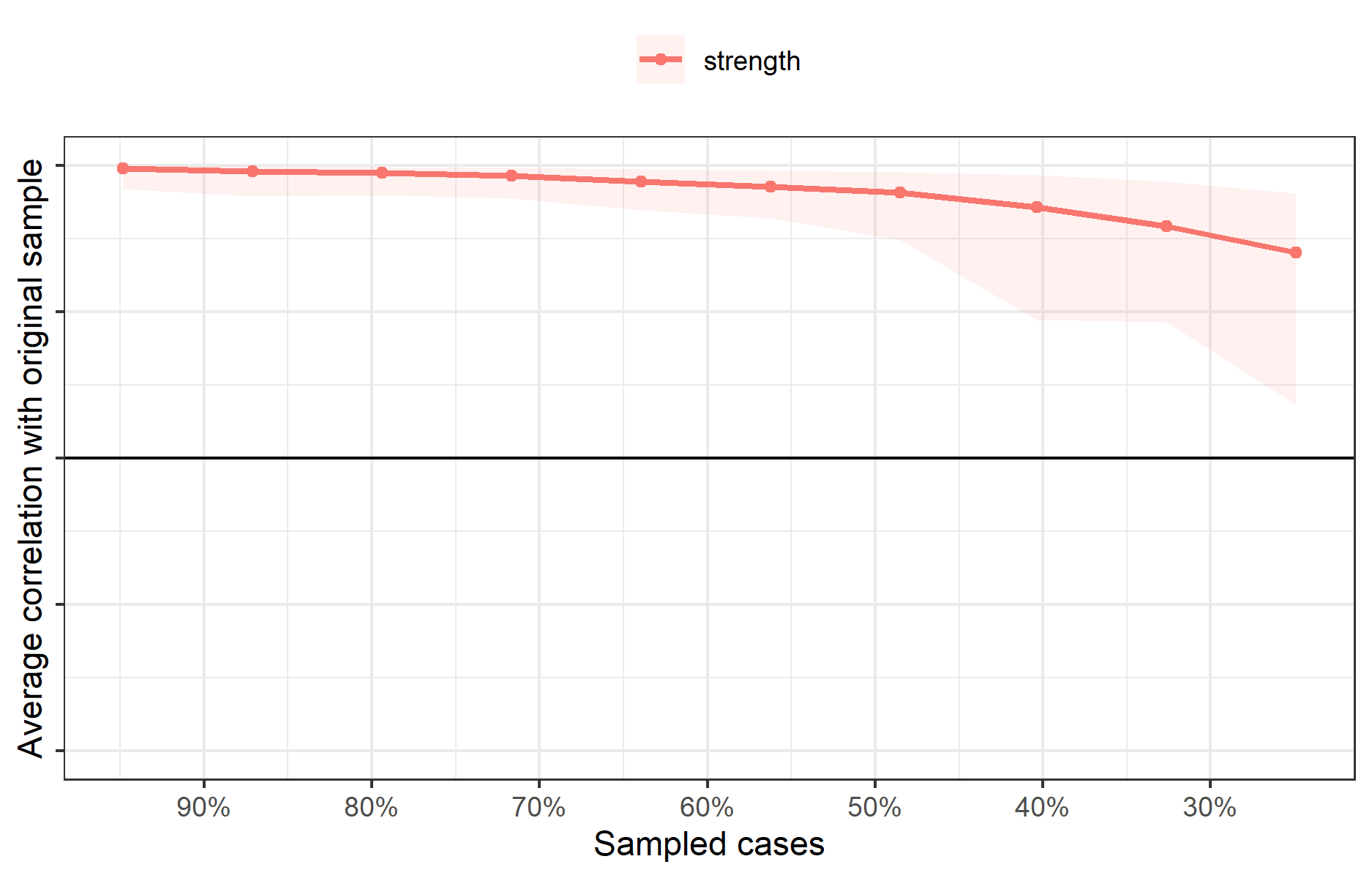

Supplement: Supplementary FIGURE S4 — Results of network stability analyses for model 2. [file Image_4.tiff]
